# Molecular Genetic Mapping of Two Complementary Genes Underpinning Fruit Bitterness in the Bottle Gourd (*Lagenaria siceraria* [Mol.] Standl.)

**DOI:** 10.3389/fpls.2019.01493

**Published:** 2019-12-18

**Authors:** Xiaohua Wu, Xinyi Wu, Ying Wang, Baogen Wang, Zhongfu Lu, Pei Xu, Guojing Li

**Affiliations:** Institute of Vegetables, State Key Laboratory for Quality and Safety of Agro-products, Zhejiang Academy of Agricultural Sciences, Hangzhou, China

**Keywords:** bottle gourd, fruit bitterness, complementary genes, genetic mapping, comparative analysis

## Abstract

Fruit bitterness is a serious problem threatening the bottle gourd (*Lagenaria siceraria* [Mol.] Standl.) industry worldwide. Previous genetic studies indicated that fruit bitterness in the bottle gourd was controlled by a pair of complementary genes. In this study, based on two non-bitter landraces “Hangzhou Gourd” and “Puxian Gourd,” each of which carries a single bitterness gene, and their derived segregation populations, we mapped the complementary genes causing fruit bitterness. Quantitative trait locus (QTL) scanning based on an F_2_ population detected two QTLs, which was *QBt.1* locating in a 17.62-cM interval on linkage group (LG)2 corresponding to a 1.6-Mb region on chromosome 6, and *QBt.2* mapped to a 8.44-cM interval on LG9 corresponding to a 1.9-Mb region on chromosome 7. An advanced bulked segregant analysis (A-BSA) well validated the QTL mapping results. Sequence-based comparative analysis showed no syntenic relationship between *QBt.1/QBt.2* and the known bitterness genes in cucumber, melon, and watermelon, suggesting that causal genes underlying *QBt.1* and *QBt.2* were not direct orthologs of the reported cucurbit bitterness genes. Our results shed light on the molecular genetic mechanisms underlying fruit bitterness in the bottle gourd and is useful to guide breeders to properly select parental lines to avoid the occurrence of bitter fruits in breeding programs.

## Introduction

Bottle gourd or calabash (*Lagenaria siceraria* [Mol.] Standl.) (2*n* = 2*x* = 22), a member of the genus *Lagenaria* of the Cucurbitaceae family (Beevy and Kuriachan, 1996), is recognized as indigenous to Africa and domesticated independently in Asia (Erickson et al., 2005). Having a cultivation history of over 8,000 years by man (Crawford, 1992), today, the bottle gourd is grown all over the tropics and subtropics for its immature fruits used as a vegetable or hard-shelled mature fruits used as containers, musical instruments, or handicrafts. The bottle gourd seedlings are widely used as a rootstock for grafting with watermelon to defend soil-borne diseases and to increase low-temperature tolerance (Davis et al., 2008; King et al., 2008).

While young fruits of bottle gourd are traditionally consumed as a delightful culinary vegetable in many areas of Asia including China, the undesirable occurrence of fruit bitterness in this crop as documented in the ancient medical books *Ben Cao Jing Ji Zhu* and *Ben Cao Gang Mu* has long been threatening the bottle gourd industry. Fruit bitterness not only affects the economic value of the bottle gourd but causes severe food poisoning symptoms such as nausea, vomiting, diarrhea, and abdominal cramps in humans and livestock (Zhang, 1981). Fruit bitterness appears to be a common trait to the Cucurbitaceae family, in which the compounds cucurbitacins having the function of defending against insects and herbivores cause the bitterness phenotype (Balkema-Boomstra et al., 2003). In cucumber, the effective cucurbitacin component is cucurbitacin C (CuC). It has been clear that nine genes are involved in the CuC biosynthetic pathway, and two genes, *Bl* (Bitter leaf) and *Bt* (Bitter fruit), regulate the bitterness/non-bitterness phenotype in leaves and fruits, respectively. The selection of natural mutations on *Bi* and *Bt* has played an important role in the domestication of ancient wild cucumber to form the present-day non-bitter cultivars (Shang et al., 2014). The major gene clusters for cucurbitacin biosynthesis were found to be highly conserved in cucumber, melon, and watermelon, while the regulatory genes seemed divergent among the three crops (Zhou et al., 2016).

Previous studies through traditional genetic analysis indicated that fruit bitterness in the bottle gourd was controlled by a pair of complementary genes (Zhang, 1981); however, the genome locations of the genes remain unknown. Genome-wide scan for quantitative trait loci (QTLs) is an effective approach for mapping traits governed by multi-genes including digenic genes (Zhang et al., 2013). Alternatively, Wu and Huang (2006) developed an advanced bulked segregant analysis (A-BSA hereafter) method to specifically identify DNA markers linked to two interactive genes. This method relies on the construction of a DNA pool mixed with homozygous genotypes (homo-pool) and another pool mixed from individuals with heterozygous genotypes showing contrasting phenotypes (heter-pool). By comparing marker genotypes among the parental lines and the two pools, DNA markers linked to the trait-determining genes as well as their parental allele origins can be inferred.

In the current study, we mapped the fruits bitterness genes in the bottle gourd genome and revealed their relationships with known bitterness genes in related food cucurbits. Our study also demonstrates the usefulness of combining QTL scanning and A-BSA as a fast and efficient approach to map and validate complementary genes controlling a trait.

## Materials and Methods

### Plant Materials and Growth Conditions

**Figure 1:**
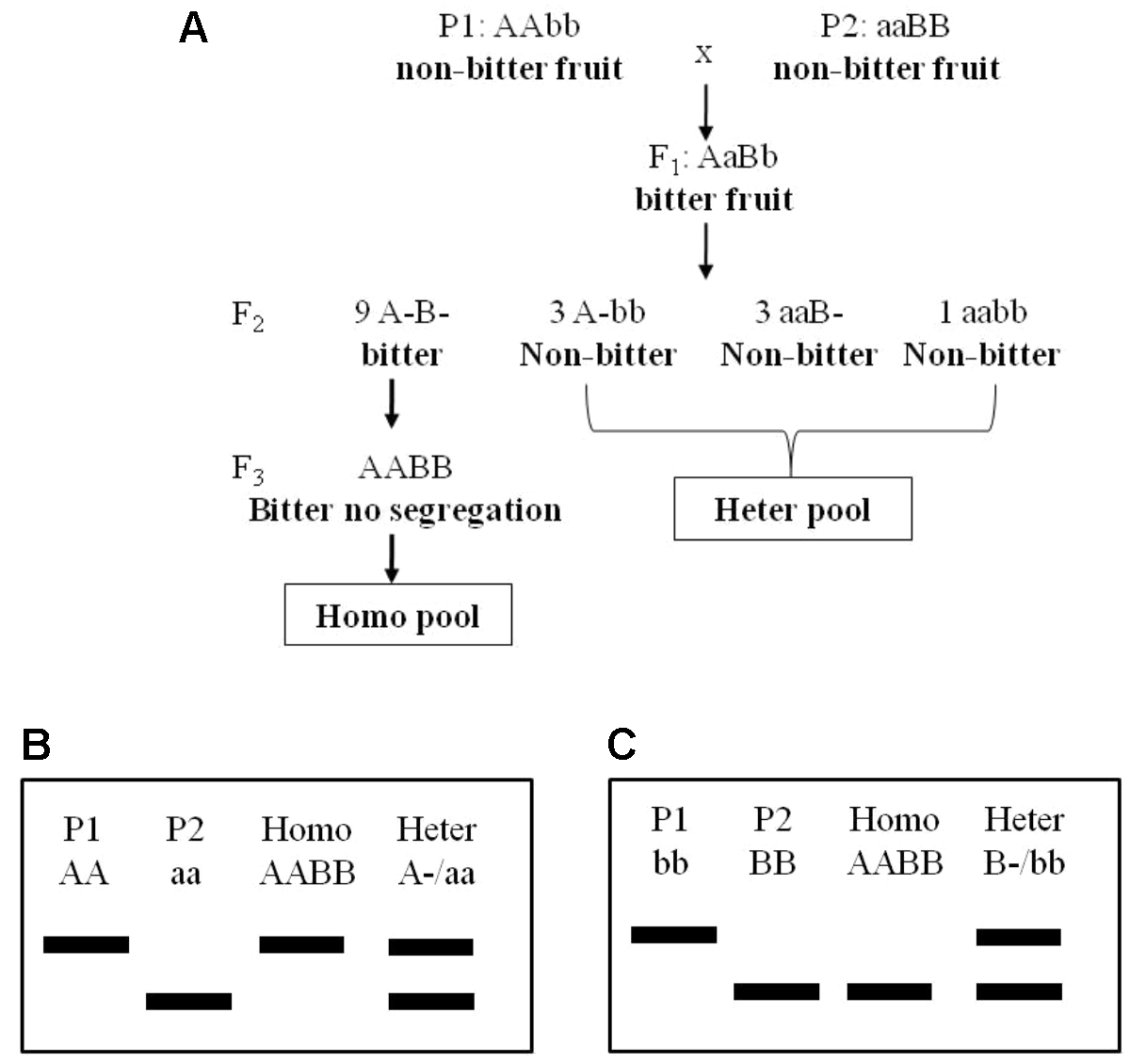
The genetic model of fruit bitterness in the F_2_ population **(A)** and advanced BSA method using in this study **(B**, **C)**. BSA, bulked segregant analysis.

Two bottle gourd landraces “Hangzhou Gourd” (HZ hereafter) and “Puxian Gourd” (PX hereafter), their F_1_ progenies, and F_2_ populations derived from selfing independent F_1_s in the years 2013, 2014, and 2016 were used in this study. The population size for the three F_2_ populations (2013F_2_, 2014F_2_, and 2016F_2_) were 102, 169, and 101, respectively. For A-BSA, 24 individuals showing a bitter-fruit phenotype from the 2013F_2_ population were screened for their F_3_ progenies phenotypes, and those showing no fruit bitterness segregation in F_3_ generation were selected to construct the homozygous bitter pool (homo-pool). Six F_2_ individuals with non-bitter fruits were randomly selected to construct a heter-pool (**Figure 1**). All plants were grown in tunnel houses in 30-m rows spaced 0.5 m apart. Ambient temperature and light as well as normal management were applied.

### Fruit Bitterness Evaluation

Fruit bitterness evaluation was conducted at the stage of 8–12 days after pollination. Bitterness phenotype of each individual was determined by manually tasting the sliced fruit sarcocarp by three trained tasters according to a classic tasting method described by Andeweg and DeBruyn (1959). The bitterness phenotype was scored as 1 for bitter and 0 for non-bitter. Only those results that were consistent among all three tasters were considered trustable and used in further analysis.

### DNA Extraction and Single-Nucleotide Polymorphism Genotyping

Genomic DNA was extracted from young leaves of 2-week-old seedlings using a DNA extraction kit (TIANGEN Co. Ltd, Beijing) following the manufacturer’s instructions. For single-nucleotide polymorphism (SNP) genotyping of the mapping populations, our previous RAD-Seq data from a set of bottle gourd accessions including HZ and PX were revisited (Wu et al., 2017a), from which 192 out of the 684 SNPs between the two parents that were evenly distributed in the genome were selected. Kompetitive allele-specific PCR (KASP) assays were used to genotype the mapping populations. KASP primers were designed using the Kraken^™^ software system (https://www.biosearchtech.com/support/tools/genotyping-software/kraken). Each KASP reaction was carried out in a final volume of 10 µl containing 20–40 ng of genomic DNA, 5 µl of 2× premade KASP master mix (LGC, Middlesex, UK), and 0.14 µl of primer mix. PCR amplification was performed in a Hydrocycler^2^ water bath thermal cycler following LGC parameters: 95°C, 15 min for hot-start Taq DNA polymerase activation, followed by a touchdown profile of 10 cycles at 94°C for 20 s and 61°C for 1 min with a 0.6°C reduction per cycle, and followed by 26 cycles at 94°C for 20 s and 55°C for 1 min. End-point fluorescent images were visualized using the BMG FLUOstar Omega (https://www.biosearchtech.com/products/instruments-and-consumables/genotyping-instruments/snpline-genotyping-automation/plate-reading), and allele calls for each genotype were obtained using the KlusterCaller^™^ software (LGC, UK).

### Linkage Mapping and Quantitative Trait Locus Analysis

A genetic linkage map for SNPs was constructed based on the 2014F_2_ population using the software QTL IciMapping (http://www.isbreeding.net). A likelihood of odds (LOD) threshold of 3.0 was used to determine the linkage groups (LGs), and an nnTwoOpt algorithm was used to determine the maker orders in each LG. The software MapQTL V5 (Van Ooijen, 2004) was used to detect bitterness QTL. Firstly, the interval mapping (IM) model was applied to detect QTLs for fruit bitterness, and then a multiple-QTL model (MQM) was used to scan for new QTLs with the markers closest to the original QTLs being implemented as cofactors. The mapping parameters were as follows: step size = 1.0, maximum number of neighboring markers = 5, maximum number of iterations = 200, and function tolerance = 1.0*e* ^−08^. A genome-wide permutation test with 1,000 random permutations was conducted to obtain an empirical LOD score threshold for significance (*P* < 0.05).

### Comparative Mapping of Bitterness Genes

The coding DNA sequence (CDS) of the bitterness genes *Bt*, *Bi*, and *Bl* from cucumber, melon, and watermelon (Shang et al., 2014; Zhou et al., 2016) were Blastn searched against the latest Hangzhou Gourd reference genome assembly V2.0 (Wang et al., 2018) to locate their syntenic region and orthologous genes in the bottle gourd genome with an *e*-value cutoff of 1*e*−10. The annotation of genes in the bottle gourd QTL intervals was retrieved from GourdBase (Wang et al., 2018).

## Results

### Inheritance of Fruit Bitterness in the Mapping Populations

Fruit bitterness assessment of parental lines, their F_1_ progenies, and F_2_ populations showed consistent phenotypes in the three biological replicates in 2013, 2014, and 2016. HZ and PX always showed a non-bitter phenotype, whereas their F_1_ progenies were always bitter. For each of the three F_2_ sub-populations, the bitter- and non-bitter-fruited individuals fit a 9:7 segregating ratio (**Table 1**), which suggested two complementary genes controlling this trait as previously reported in other cultivars of this species (Zhang, 1981).

**Table 1:** Fruit bitterness phenotypes.

Genotype/population	Number of individuals			
	Bitter	Non-bitter	χ^2^	Ratio
Hangzhou Gourd	0	48		
Puxian Gourd	0	48		
F_1_	48	0		
2013F_2_	59	43	0.16	9:7
2014F_2_	106	63	2.65	9:7
2016F_2_	63	38	1.37	9:7

### Quantitative Trait Locus Mapping for Fruit Bitterness

**Figure 2:**
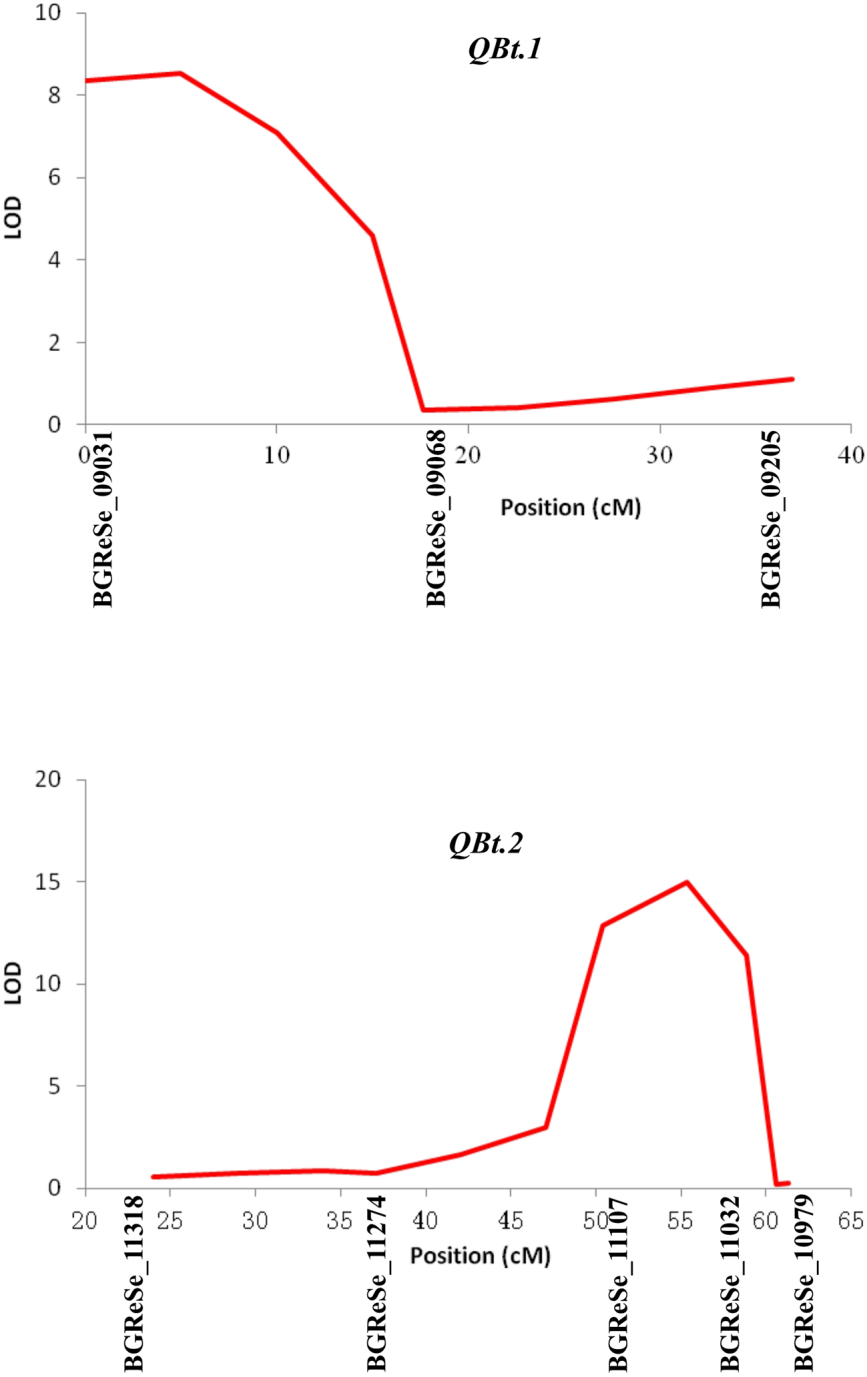
QTLs detected in the F_2_ population under a multiple-QTL model using MAPQTL V5. QTL, quantitative trait locus.

Of the 192 intended KASP assays, 174 successfully detected signals and 153 called polymorphic genotypes in the F_2_ population in 2014. Based on these 153 SNPs data, a genetic linkage map containing 147 SNP loci distributed on 11 LGs were constructed. In line with the digenic mode of fruit bitterness inheritance in this population, two QTLs, designated as *QBt.1* and *QBt.2*, were detected through IM followed by MQM (**Table 2**, **Figure 2** and **Supplementary Table 1**). *QBt.1* was located in a 17.62-cM interval between the SNP markers BGReSe_09031 and BGReSe_09068 on LG2, which explained 18% of the phenotype variance; *QBt.2* was mapped to an 8.44-cM interval defined by the SNP markers BGReSe_11107 and BGReSe_11032 on LG9, which accounted for 27.7% of the phenotype variance. QTL epistatic interaction was detected between *QBt. 1*and *QBt.2*, and the phenotypic variation explained reached 99.32%, indicating that *QBt.1*and *QBt.2* were the pair of complementary genes controlling fruit bitterness in this population.

**Table 2:** QTLs for fruit bitterness detected in the 2014 F_2_ population.

QTL	LG	Flanking marker	Genetic position	LOD	*R* ^2^ (%)
*QBt.1*	LG2	BGReSe_09031-BGReSe_09068	0–17.62	8.35	18
*QBt.2*	LG9	BGReSe_11107-BGReSe_11032	50.37–58.81	12.86	27.7

QTL, quantitative trait locus; LG, linkage group.

### Advanced Bulked Segregant Analysis for Single-Nucleotide Polymorphisms Linked to Fruit Bitterness

Another mapping approach, A-BSA, was also applied to screen for DNA markers linked to fruit bitterness. We screed 192 genome-wide distributed SNPs among the two parental lines, the homo-pool and the heter-pool, by using the KASP technology. It turned out that the marker BGReSe_09068 showed identical homozygous genotypes between HZ and the homo-pool, while it exhibited an alternative homozygous genotype in PX and, as expected, heterozygous genotype in the heter-pool (**Table 3**). Likewise, the marker BGReSe_11107 showed identical homozygous genotype in PX and the homo-pool but alternative homozygous genotype in HZ and heterozygous genotype in the heter-pool (**Table 3**). These results suggested that BGReSe_09068 and BGReSe_11107 each were linked to one of the complementary genes in HZ and PX, respectively. Coincidently, these two markers were found to fall into the QTLs intervals of *QBt.1* and *QBt.2*, respectively. These results thus provide strong validation for the QTL mapping result and indicates that *QBt.1* is one of the bitterness gene alleles from HZ and *QBt.2* is the other from PX.

**Table 3:** The genotypes of linked markers detected using the advanced BSA method.

SNP	Hangzhou Gourd	Puxian Gourd	Homo-pool	Heter-pool
BGReSe_09068	TT	CC	TT	TC
BGReSe_11107	TT	CC	CC	TC

BSA, bulked segregant analysis; SNP, single-nucleotide polymorphism.

### Comparative Analysis of Bitterness Loci in Major Cucurbit Crops

**Figure 3:**
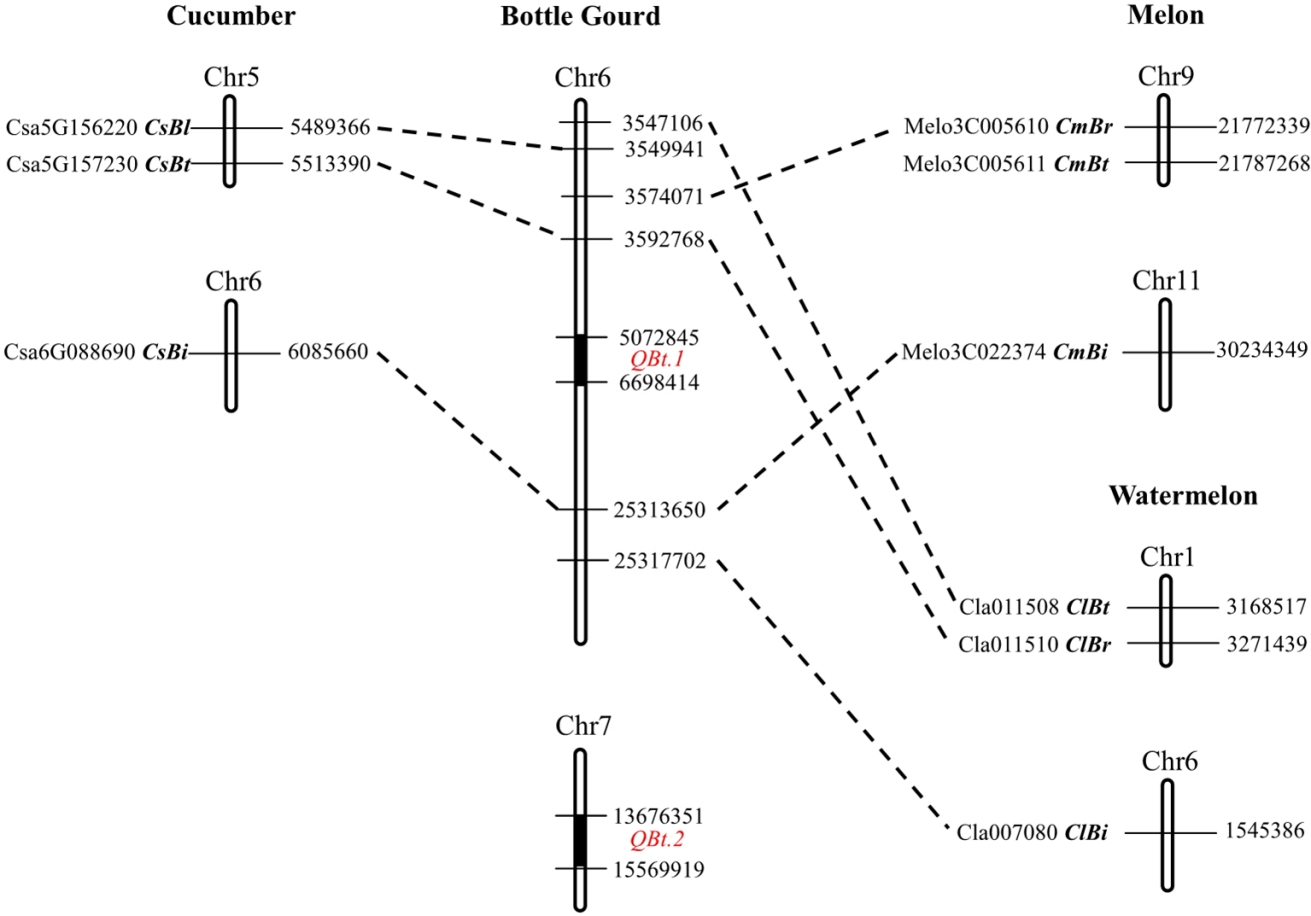
Genomic synteny of the bitterness genes between bottle gourd, cucumber, melon. and watermelon.

According to the physical locations of the QTL-flanking markers on the HZ reference genome V2.0, *QBt.1* resides in a 1.62-Mb region (5,072,845–6,698,414 bp) on chromosome 6 with 142 predicted genes, and *QBt.2* in a 1.89-Mb region (13,676,351–15,569,919 bp) containing 89 predicted genes on chromosome 7. To elucidate the relationship between the bottle gourd fruit bitterness QTLs and the known bitterness genes in related cucurbits, a cross-mapping analysis was performed. It showed that the cucumber bitterness genes *CsBt* and *CsBi* and the *Bi* regulator gene *CsBl* were syntenic to the genomic region on chromosome 6 in the bottle gourd (**Figure 3**, **Supplementary Table 2**). The four tissue-specific cucurbitacin regulator genes (*CmBt* and *CmBr*, *ClBt* and *ClBr*) in melon and watermelon were also found to be syntenic to the same region on chromosome 6 in bottle gourd except for *CmBt*. *CmBi* in melon and *ClBi* in watermelon found their orthologous genes in the distal region on chromosome 6 in bottle gourd (**Figure 3**, **Supplementary Table 2**). However, neither *QBt.1* nor *QBt.2* was located in or close to the syntenic region of these known bitterness genes. In addition, all the cucurbitacin biosynthetic genes in cucumber, melon, and watermelon found orthologs in the bottle gourd genome, but none of them were located in the QTL regions (**Supplementary Table 2**). These results suggest that the causal genes underlying *QBt.1* and *QBt.2* are unlikely to be direct orthologs of the bitterness genes in these related cucurbit crops.

## Discussion

It has long been known that fruit bitterness in the bottle gourd is controlled by two interacting genes with complementary effect (Zhang, 1981). However, due to the lack of genomic resources, genetic mapping and elucidation of the bitterness genes in this species lag behind. Recently, plenty of SSR, Indel, and SNP markers information for the bottle gourd were released (Xu et al., 2011; Wu et al., 2017b; Wang et al., 2018), and reference genomes were available to the public (Xu et al., 2014; Wu et al., 2017c; Wang et al., 2018), allowing for more in-depth characterization of bitterness genes in this species. In this study, we initially mapped two QTLs, *QBt.1* and *QBt.2*, each to a less than 2-Mb segment on chromosome 6 and chromosome 7, respectively. Then, by applying an advanced BSA method, we validated the QTL mapping results and elucidated that the functional alleles of *QBt.1* and *QBt.2* were from HZ and PX, respectively.

The ancestor wild cucumber, melon, watermelon, and bottle gourd plants all exhibited an extreme bitter phenotype in fruits (Zhou et al., 2016). Common bitterness compounds and their biosynthetic pathways were found in these related cucurbit crops (Lester, 1997; Matsuo et al., 1999; Chen et al., 2005). Likely due to common human demands for fruit quality, the domestication roadmap from extremely bitter wild ancestor to non-bitter modern cultivars seems to be also similar among cucumber, melon, and watermelon (Zhou et al., 2016). It has been argued that the causative mutations at orthologous genes may underlie convergent changes in fruit bitterness in cucurbits. For example, the loss of fruit bitterness during domestication in cucumber, melon, and watermelon all were caused by mutations on *Bt* genes (*CsBt*, *ClBt*, and *CmBt*) that are syntenic between genomes (Zhou et al., 2016). The cucumber *CsBt* gene encodes a basic helix–loop–helix (bHLH) transcription factor that activates the foliar bitterness gene *Bi* and regulates CuC biosynthesis in the fruit (Shang et al., 2014). According to the HZ reference genome V2.0, a gene *HG_GLEAN_10009202* was found to be the putative orthologous gene of *Bt*; however, this gene does not fall into the QTL intervals of *QBt.1* nor *QBt.2*. In addition, the bottle gourd orthologs of the cucumber foliar bitterness genes *CsBi* and *CsBl*, and the melon and watermelon root bitterness genes *CmBr* and *ClBr*, were all found to be not coincident with *QBt.1* or *QBt.2*. Therefore, the causal genes underlying the bitterness QTLs in the bottle gourd are unlikely to be direct orthologs of the cucumber *CsBt*, *CmBr*, or *ClBr* gene. However, this result does not exclude the possibility that similar genes or same gene family members are the bitterness gene in the bottle gourd. Of the 89 predicted genes in the *QBt.2* region, there is a transcription factor bHLH35-like isoform X1 gene and three cytochrome P450 CYP73A100-like genes, which might be functionally related to known cucurbit bitterness genes. More future work is required to draw a clearer picture on the causal genes underlying bitterness QTLs in the bottle gourd.

Our results shed new light on the molecular genetic mechanisms underlying fruit bitterness in the bottle gourd. From the perspective of breeding, it will be useful to guide the selection of parental lines to avoid the occurrence of bitter fruits in breeding programs. Breeders could use the flanking markers to *QBt.1* and *QBt.2* to screen their germplasm and breeding lines to predict if the hybrids would show a bitterness phenotype without the need of making real crosses.

## Data Availability Statement

All datasets generated for this study are included in the article/**Supplementary Material**.

## Author Contributions

XYW, PX and GL conceived and designed the research. XYW performed the experiments and wrote the manuscript. XHW constructed the population and collected bitterness phenotypes. YW, BW and ZL carried out the field work. All authors analyzed the data and read and approved the final manuscript. PX revised the manuscript.

## Funding

This study was partially supported by the National Natural Science Foundation of China (31401880), the Major Science and Technology Project of Plant Breeding in Zhejiang Province (2016C02051), and the project from State Key Laboratory for Quality and Safety of Agroproducts (2010DS700124-ZZ1808).

## Conflict of Interest

The authors declare that the research was conducted in the absence of any commercial or financial relationships that could be construed as a potential conflict of interest.

## Supplementary Material

SUPPLEMENTARY TABLE 1 Genotype data and phenotype data for QTL mapping in the F_2_ population.

SUPPLEMENTARY TABLE 2 Colinearity analysis of the cucurbitacin biosynthetic and regulatory genes in four cucurbits.
