# Supplementary material for: Molecular Genetic Mapping of Two Complementary Genes Underpinning Fruit Bitterness in the Bottle Gourd (Lagenaria siceraria [Mol.] Standl.)
Source: Front Plant Sci. 2019 Dec 18;10:1493. doi: 10.3389/fpls.2019.01493 (PMC6930244; doi:10.3389/fpls.2019.01493)
Supplement: SUPPLEMENTARY TABLE 2 — Colinearity analysis of the cucurbitacin biosynthetic and regulatory genes in four cucurbits. [file Table_2.doc]

Supplementary Table 2 Colinearity analysis of the cucurbitacin biosynthetic and regulatory genes in four cucurbits.

| Cucumber | |  | Melon | |  | Watermelon | |  | Bottle gourd | |
| --- | --- | --- | --- | --- | --- | --- | --- | --- | --- | --- |
| Abbr. | Gene ID |  | Abbr. | Gene ID |  | Abbr. | Gene ID |  | Genomic region | Gene ID |
| Biosynthetic genes |  |  |  |  |  |  |  |  |  |  |
| *Cs160* | *Csa6G088160* |  | *Cm160* | *Melo3C022377* |  | *Cl160* | *Cla007077* |  | Chr06:25338074 to 25339030 | no hits |
| *Cs170* | *Csa6G088170* |  | *Cm170* | *Melo3C022376* |  | *Cl170* | *Cla007078* |  | Chr06:25334712 to 25333179 | *HG_GLEAN_10010733* |
|  |  |  | *Cm180* | *Melo3C022375* |  | *Cl180* | *Cla007079* |  | Chr06:25324197 to 25325084 | no hits |
| *CsBi* | *Csa6G088690* |  | *CmBi* | *Melo3C022374* |  | *ClBi* | *Cla007080* |  | Chr06:25313649 to 25313955 | *HG_GLEAN_10010732* |
| *CsACT* | *Csa6G088700* |  | *CmACT* | *Melo3C022373* |  | *ClACT* | *Cla007081* |  | Chr06:25296603 to 25297925 | *HG_GLEAN_10010731* |
| *Cs710* | *Csa6G088710* |  | *Cm710* | *Melo3C022372* |  | *Cl710* | *Cla007082* |  | Chr06:25289017 to 25288617 | *HG_GLEAN_10010730* |
| *Cs890* | *Csa1G044890* |  | *Cm890* | *Melo3C002192* |  | *Cl890A* | *Cla008355* |  | Chr05:2438252 to 2438587 | *HG_GLEAN_10020786* |
|  |  |  |  |  |  | *Cl890B* | *Cla008354* |  | Chr05:2438252 to 2438587 | *HG_GLEAN_10020786* |
| *Cs490* | *Csa3G698490* |  | *Cm490* | *Melo3C023960* |  | *Cl490* | *Cla017252* |  | Chr04:21817654 to 21816995 | *HG_GLEAN_10019434* |
|  |  |  |  |  |  | *Cl510* | *Cla016164* |  | Chr01:1333991 to 1333512 | *HG_GLEAN_10010988* |
| *Cs540* | *Csa3G903540* |  |  |  |  |  |  |  | Chr01:1260914 to 1261035 | *HG_GLEAN_10010985* |
| *Cs550* | *Csa3G903550* |  |  |  |  |  |  |  | Chr01:1236926 to 1237406 | *HG_GLEAN_10010983* |
| Regulatory genes | |  |  |  |  |  |  |  |  |  |
| *CsBl* | *Csa5G156220* |  |  |  |  |  |  |  | Chr06:3550250 to 3549941 | *HG_GLEAN_10009202* |
| *CsBt* | *Csa5G157230* |  |  |  |  | *ClBr* | *Cla011510* |  | Chr06:3593185 to 3592768 | *HG_GLEAN_10009210* |
|  |  |  | *CmBr* | *Melo3C005610* |  |  |  |  | Chr06:3574510 to 3574071 | *HG_GLEAN_10009209* |
|  |  |  | *CmBt* | *Melo3C005611* |  |  |  |  | no hits | no hits |
|  |  |  |  |  |  | *ClBt* | *Cla011508* |  | Chr06:3547410 to 3547106 | *HG_GLEAN_10009208* |

Abbr, abbreviation. The gene abbreviation in cucumber, melon and watermelon was cited from Zhou et al. (2016). Orthologous genes are listed in rows.
